# High hydrostatic pressure enhanced the growth of deep-sea *Thermococcus aciditolerans* by promoting the reduction of elemental sulfur

**DOI:** 10.3389/fmicb.2025.1643593

**Published:** 2025-08-18

**Authors:** Ze-Xi Jiao, Xue-Gong Li, Wei-Jia Zhang, Guan-Yuan Zhang, Shi-Jie Bai, Ling Fu, Long-Fei Wu

**Affiliations:** ^1^Laboratory of Deep-Sea Microbial Cell Biology, Institute of Deep-Sea Science and Engineering, Chinese Academy of Sciences, Sanya, China; ^2^College of Marine Sciences, University of Chinese Academy of Sciences, Beijing, China; ^3^International Associated Laboratory of Evolution and Development of Magnetotactic Multicellular Organisms, CNRS-Marseille/CAS, Sanya, China; ^4^Hainan Deep-Sea Technology Laboratory, Institution of Deep-Sea Life Sciences, IDSSE-BGI, Sanya, Hainan, China; ^5^Laboratory of Marine Viruses and Molecular Biology, Institute of Deep-Sea Science and Engineering, Chinese Academy of Sciences, Sanya, China; ^6^State Key Laboratory of Proteomics, Beijing Proteome Research Center, National Center for Protein Sciences Beijing, Beijing Institute of Lifeomics, Beijing, China; ^7^CNRS, LCB, IMM, IM2B, Aix Marseille University, Marseille, France

**Keywords:** *Thermococcus aciditolerans* SY113, high hydrostatic pressure, elemental sulfur reduction, membrane-bound sulfane reductase, membrane-bound hydrogenase, SurR

## Abstract

*Thermococcus* species are ubiquitously distributed across both shallow and deep-sea hydrothermal vent ecosystems. Elemental sulfur (S°) reduction plays a pivotal role in their energy metabolism. While extensive characterization of the MBS and MBH pathways, along with their SurR-dependent regulatory network, has been established in shallow-water model strains, understanding of the high hydrostatic pressure (HHP) and sulfur-responsive regulation of these pathways in deep-sea *Thermococcus* lineages remains limited. In this study, we investigated the effects of HHP on both growth and S° reduction in the deep-sea SY113 strain, as well as its regulatory impact on *mbs* and *mbh* expression. Our results demonstrate that HHP enhances both S° reduction and growth in SY113 strain, independent of the general regulator SurR. Genetic disruption of *mbsL* significantly impaired H_2_S production and growth under HHP conditions, establishing the essential role of S° reduction in HHP adaptation. Furthermore, disrupted *mbhL1* gene confirmed that a single MBS complex is sufficient to maintain pressure-stimulated growth. The gene expression analysis revealed that the expression of *mbsL* gene is primarily promoted by S°, while the expression of *mbhL1* gene is induced by HHP. Moreover, the expression of these genes exhibits correlation. Additionally, we found that the expression of *mbsL* gene, *mbhL1* gene, and *mbhL2* gene in SY113 strain is not only regulated by SurR, and HHP also plays a role in modulating the expression of these genes. Overall, the sulfur responsive regulation of gene expression in SY113 strain distinguishes from that in the shallow model strains, which implies an adaptive strategy for *Thermococcus* species used to dwell in the deep-sea hydrothermal vent.

## 1 Introduction

The order Thermococcales is widely distributed across geothermal habitats, including shallow and deep-sea hydrothermal vents, as well as high-temperature oil reservoirs (Schut et al., 2014). It consists of three genera, *Pyrococcus* (Fiala and Stetter, 1986), *Thermococcus* (Zillig et al., 1983) and *Paleococcus* (Takai et al., 2000), these microorganisms represent deep evolutionary branches within the domain Archaea (Stetter, 2006; Canganella and Wiegel, 2014). Their phylogenetic position makes them particularly valuable for understanding early stages of unicellular life evolution.

Members of the Thermococcales order are hyperthermophilic, anaerobic heterotrophs that utilize peptides, amino acids, and oligosaccharides as carbon sources, while employing protons or S° as terminal electron acceptors to produce molecular hydrogen (H_2_) or hydrogen sulfide (H_2_S) (Leigh et al., 2011; Schut et al., 2014). While S^0^ generally enhances growth and is essential for certain Thermococcales species, it's extremely low solubility necessitates conversion to biologically accessible forms. Cyclooctasulfur is the main form of S^0^ and its S_8_ ring reacts readily with H_2_S to form soluble polysulfide (Wu et al., 2018). These polysulfides serve as substrates for the membrane-bound sulfane reductase (MBS), first characterized in *Pyrococcus furiosus* (Schut et al., 2007). The MBS complex catalyzes the reduction of organic and anionic polysulfides using reduced ferredoxins as electron donors, ultimately yielding H_2_S through spontaneous disproportionation of the resulting small anionic polysulfides (Wu et al., 2018).

MBS is encoded by 13 genes (*mbsABCDEGHH'MJKLN*) and composed of a transmembrane MbsABCDEGHH'M complex and a cytoplasmic MbsJKLN module. MbsM anchors the cytoplasmic module to the transmembrane module. Among them, the MbsJKLN module transfers the electrons from the ferredoxin to the substrate polysulfide with the MbsL as the catalytic subunit (Schut et al., 2013; Wu et al., 2018; Yu et al., 2020, 2021). In the transmembrane module, both MbsH and MbsH' subunits function as H^+^ pumps that transport protons out of the membrane and creates a proton gradient. The MbsABCDEG transmembrane sub-complex contains some H^+^/Na^+^-translocating units that generate a sodium ion gradient across the cell membrane using the proton gradient. The Na^+^-driven ATP synthase uses the created sodium ion gradient to produce ATP (Silva et al., 2000; Sapra et al., 2003; Wu et al., 2018). In Thermococcales genome, there is also a 14-gene cluster termed membrane-bound hydrogenase complex (MBH), which is highly homologous to MBS (Schut et al., 2013; Yu et al., 2018, 2021). The most obvious difference between them is MBH has additional cysteine residues in the [NiFe] binding motif of its catalytic subunit MbhL, and could use reduced ferredoxins to reduce proton, following H_2_ production (Schut et al., 2013; Yu et al., 2018). During this reaction, reducing equivalents are disposed of as H_2_, coupled with the generation of a sodium ion gradient to produce ATP (Sapra et al., 2003; Kanai et al., 2011; Schut et al., 2013). In addition, MBH contains only a single proton pump (MbhH), which means that MBH may conserve less energy than MBS.

Studies on shallow water model strains *P. furiosus* and *Thermococcus kodakarensis* demonstrated that the MBS and MBH were antagonistically regulated by the addition of S^0^ through a sulfur-dependent redox switch SurR (Lipscomb et al., 2009; Yang et al., 2010; Santangelo et al., 2011; Lipscomb et al., 2017). The DNA binding activity of SurR is dependent on the redox status of a CxxC motif located in its N-terminal region. In the absence of S^0^, SurR with reduced CxxC motif could bind to the target DNA sequence “GTTn_3_AAC” (Yang et al., 2010). This DNA motif is present in the promoter regions of both *mbs* and *mbh*. It is located upstream of the BRE/TATA element in the *mbh* promoter region, but overlap with the BRE/TATA element of *mbs* (Hidese et al., 2017). Consequently, SurR binding to the “GTTn_3_AAC” motif activates transcription of the *mbh* but prevents RNA polymerase recruitment and access of the transcription apparatus to the promoter, thus inhibit the transcription of *mbs*. In the presence of S^0^, the cysteine residues of the CxxC motif are oxidized and the oxidized SurR loses its capacity to bind the “GTTn_3_AAC” motif. Therefore, transcription of *mbh* is repressed, while *mbs* is de-repressed (Lipscomb et al., 2009; Yang et al., 2010).

At the time of writing, the order Thermococcales comprises 48 species with valid published names (https://lpsn.dsmz.de/order/thermococcales). Among them, 39 species belong to the genus *Thermococcus*, including 27 species isolated from deep-sea hydrothermal vents. Although most *Thermococcus* strains have been isolated from deep-sea hydrothermal vents, very little is known about how they adapt to the HHP. Studies of deep-sea strains of *Pyrococcus yayanosii* (Michoud and Jebbar, 2016), *Thermococcus barophilus* (Vannier et al., 2015) and *Thermococcus piezophilus* (Moalic et al., 2021) showed that HHP regulated the expression of genes associated with some metabolic pathways, especially pathways involved in energy production. Moreover, recent studies on *T. barophilus* have revealed that SurR-mediated regulation of energy metabolism genes is influenced by both sulfur availability and HHP (Moalic et al., 2025). In this study, we selected *Thermococcus aciditolerans* SY113, isolated from a deep-sea hydrothermal vent chimney on the Southwest Indian Ridge at a depth of 2,770 m (Li et al., 2021), as a representative deep-sea *Thermococcus* strain. We focused on the S^0^ reduction in connection with MBS and MBH, and the HHP regulation of the expression of these enzyme complexes. Here we show that HHP improves the S^0^ reduction and growth of SY113 strain. Disruption of *mbsL* gene impairs the H_2_S production and growth of SY113 strain under HHP, which indicates that S^0^ reduction plays an important role in the HHP tolerance. Furthermore, disruption of the *mbhL1* gene resulted in increased growth rate, maximum biomass, and H_2_S production under HHP in the mutant strain compared to the wild type, further supporting the role of elemental sulfur reduction in enhancing HHP tolerance. Gene expression analysis revealed that SurR regulates *mbs* and *mbh* operons in SY113 strain in a manner distinct from shallow-water model strains. Notably, this SurR-mediated regulation exhibits conserved features with the deep-sea model strain *T. barophilus* MP (Moalic et al., 2025). Furthermore, HHP was identified as an additional regulatory factor influencing these operons. Collectively, these results reveal the adaptive strategy of strain SY113 to deep-sea hydrothermal vent environments, expanding our understanding of the regulatory mechanisms governing elemental sulfur metabolism in deep-sea *Thermococcus* species.

## 2 Materials and methods

### 2.1 Strains and growth conditions

*T. aciditolerans* SY113 was cultivated under anaerobic conditions in TRM medium at 85 °C (Zeng et al., 2009; Li et al., 2021). To investigate the growth of SY113 strain, culture was grown in TRM medium supplemented with S^0^ until the stationary phase, with OD_600nm_ values ranging from 0.5 to 0.6. Then the culture was centrifuged at 2,000 × *g* and 4°C for 10 min to remove sulfur powder, transfer the supernatant liquid to a new tube and centrifuged again at 5,000 × *g* and 4°C for 15 min. The cell pellet was collected and washed three times with fresh anoxic TRM medium in an anaerobic chamber. The cells were then resuspended in anaerobic TRM medium to an OD_600nm_ of ~1.0. The resuspended cells were inoculated into TRM medium at 1% (v/v), then dispensed into 11 ml serum bottles in an anaerobic chamber with complete liquid filling (no headspace). For sulfur-supplemented culture conditions, elemental sulfur was added at a final concentration of 2 g/l. High-pressure cultivation at 27 MPa was performed in a stainless steel pressure vessel (Nantong Feiyu Petroleum Technology Development Co., Ltd., China) equipped with a pressure gauge and an automated water-pumping system for pressurization control. For colony selection, the TRM medium was solidified with 1% (w/v) Gelzan CM (Sigma-Aldrich, CAS 71010-52-1), and polysulfide (10 g Na_2_S·9H_2_O was dissolved in 15 ml deionized water, followed by addition of 3 g sublimed sulfur powder) was used as a substitute for elemental sulfur at a final concentration of 10 μl/ml. Under these conditions, visible colonies typically developed within 2–3 days at 85 °C.

### 2.2 RNA extraction, cDNA synthesis and quantitative real-time PCR

For RNA extraction, ~3 × 10^9^ cells in the mid-exponential growth phase were harvested by centrifugation at 8,000 × g for 20 min. Total RNA was extracted with a TRI reagent-RNA/DNA/protein isolation kit (Molecular Research Center, Inc.) according to the manufacturer's instructions, yielding ~20–30 μg of RNA per sample. For RNA purification and cDNA synthesis, the PrimeScript^TM^ II 1st strand cDNA synthesis kit (TAKARA, Shiga, Japan) was used. The RT-PCR was performed as described previously (Li et al., 2018). The expression levels of target genes were normalized to the reference gene *por* (pyruvate/ketoisovalerate ferredoxin oxidoreductase subunit gamma, POR, FPV09_09760). Each experiment was performed in triplicate for each sample, and a mean value and standard deviation were calculated for the relative RNA expression levels.

The absolute copy number of target genes was quantified by correlating the cycle threshold (Ct) values with a standard curve. The genes *mbsL, mbhL1*, and *mbhL2* from SY113 strain were cloned into the pMD™19-T vector (TAKARA, Shiga, Japan). The recombinant plasmids were transformed into *E. coli* DH5α competent cells and verified by DNA sequencing. Plasmids containing *mbsL, mbhL1*, and *mbhL2* were extracted and quantified using a NanoDrop 2000/2000c spectrophotometer (Thermo Scientific, Shanghai, China). Real-time quantitative PCR (qPCR) was conducted on a QuantStudio 3 system (Applied Biosystems). Gradient-diluted plasmids were used as templates to generate Ct values for the construction of standard curves, which were subsequently used to calculate the absolute copy numbers of the target genes.

### 2.3 Construction of gene deletion mutant

To overcome the limitations of available genetic markers, gene deletions were performed by replacing the target gene with the *3-hydroxy-3-methylglutaryl coenzyme A* (HMG-CoA) reductase gene, which confers simvastatin resistance (Sim^R^) as a positive selection marker. The Sim^R^ gene cassette was derived by fusing the promoter regions of glutamate dehydrogenase (PF1602) from *P. furiosus* DSM 3638 and HMG-CoA reductase (PYCH_01180) from *P. yayanosii* CH1 (Li et al., 2014). For counter-selection, an HHP-responsive toxin-antitoxin cassette (TAC) was utilized. The toxin gene is controlled by a HHP-inducible promoter, while the antitoxin gene is constitutively expressed under the P_*gdh*_ promoter to minimize basal toxin activity and eliminate background growth (Song et al., 2019).

Using the SY113 genome as a template, ~1 kb upstream and downstream regions of the *mbsL, mbhL1*, and *surR* genes were amplified using the primer pairs 04690-up-F/R, 03665-up-F/R, and 08560-up-F/R, respectively (Supplementary Table 1). The Sim^R^ cassette was amplified from the pUSW01 plasmid using primers P_*gdh*_-F and HMG-CoA-R, and the HHP-TAC cassette was amplified using primers hhp-tac-F/R (Supplementary Table 1). The upstream and downstream fragments, along with the Sim^R^ and HHP-TAC cassette, were assembled into a linearized pUC18 vector containing 15 bp overlapping sequences using the ClonExpress^®^ Ultra One Step Cloning Kit (Vazyme). The resulting suicide plasmids-pUS04690, pUS03665, and pUS08560-were introduced into strain SY113 via CaCl_2_-mediated transformation method (Li et al., 2014), with a transformation efficiency of ~3.3 × 10^3^ CFU per μg of plasmid DNA. Transformants were initially plated on solid TRM medium supplemented with 10 μM simvastatin and incubated at 85 °C for 2–3 days. Single colonies were then transferred to 5 ml of liquid TRM medium and incubated at 85 °C for 12–16 h. Take 500 μl of the culture and inoculate it into 50 ml of TRM medium. After incubating at 85 °C for 12–16 h, harvest the cells for genomic DNA extraction. Subsequently, verify the transformants by PCR amplification. Verified transformants were then inoculated into syringes and incubated in TRM under 27 MPa for 12 h to facilitate mutant selection via secondary homologous recombination. Following high-pressure incubation, cultures were plated onto solid TRM medium supplemented 10 μM simvastatin to isolate mutants resulting from secondary homologous recombination. The Hungate tube were incubated at 85 °C for 2–3 days. For each mutant strain, we typically selected 30–50 single colonies. These were subjected to liquid culture, followed by genomic DNA extraction, PCR amplification, and restriction enzyme digestion verification to obtain the gene knockout mutants. Overall, it takes ~3 weeks from vector construction to finally obtaining the mutant strain.

### 2.4 Quantification of H_2_S and H_2_

For quantitative determination of H_2_S, 100 μl of culture from the stationary phase was mixed with 1 ml of H_2_S absorption solution, and centrifuged at 12,000 × g for 10 min at 4 °C to remove the supernatant. Subsequently, 375 μl of N, N-dimethyl-p-phenylenediamine, and 375 μl ferric ammonium sulfate solutions were added, thoroughly mixed, and incubated at room temperature for 10 min. The absorbance was measured at 665 nm using a UV spectrophotometer. The specific H_2_S concentration measurement followed the protocol of the H_2_S assay kit (Solarbio, BC2050). The H_2_S production yield was determined by normalizing to OD_600nm_-measured biomass, using the formula: [H_2_S] sample—[H_2_S] blank control/OD_600nm_, where [H_2_S] represents the H_2_S concentration measured by methylene blue method.

For quantitative determination of H_2_, freshly prepared TRM medium was inoculated to an initial OD_600nm_ of ~0.01. Then, 5 ml of inoculated culture was dispensed into 15 ml Hungate anaerobic tubes with N_2_ as the headspace gas. The tubes were incubated at 85 °C until the stationary phase was reached. Headspace gas samples were collected from the top of the sealed anaerobic tubes to quantify H_2_ production. Gas analysis was performed using a gas chromatograph (Trace GC Ultra, Thermo Scientific) equipped with a thermal conductivity detector (TCD) set at 250 °C. Chromatographic separation was achieved using a CBP-01 column (6 ft [1.83 m] × 1/8 inch), with the injector and column oven temperatures set at 200 and 50 °C, respectively. Nitrogen served as the carrier gas at a flow rate of 20 ml/min, and the injection volume was 0.1 ml. The H_2_ production yield was determined by normalizing to OD_600nm_-measured biomass.

## 3 Results

### 3.1 HHP enhance the growth and S^0^ reduction of SY113 strain

**Figure 1:**
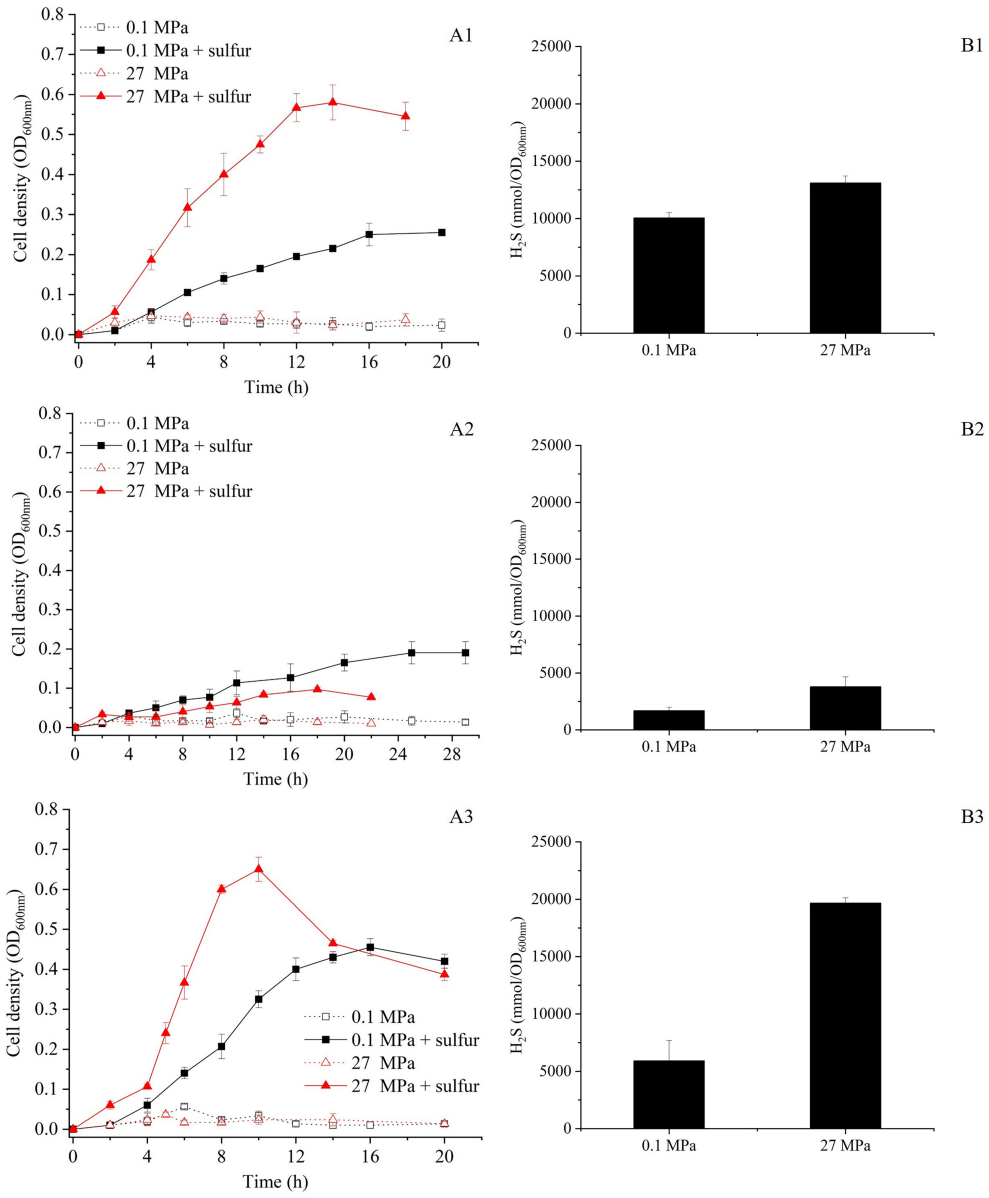
Growth curve and H_2_S production of the SY113 strain and its Δ*mbsL* and Δ*mbhL1* mutants. **(A1–A3)** Growth curves of SY113 **(A1)**, Δ*mbsL* **(A2)**, and Δ*mbhL1* **(A3)** cultured at 0.1 MPa and 27 MPa in the presence or absence of elemental sulfur, measured by OD_600nm_ over time. **(B1–B3)** Corresponding H_2_S concentrations for the SY113 strain **(B1)**, Δ*mbsL* **(B2)**, and Δ*mbhL1* **(B3)** measured at stationary phase. Black bars represent cultures with sulfur supplementation, error bars represent the standard deviation of three biological replicates. No H_2_S was produced in WT, Δ*mbsL* and Δ*mbhL1* mutants in the absence of elemental sulfur.

To investigate the effects of HHP on the growth and S^0^ metabolism, SY113 strain was cultured at 0.1 MPa and 27 MPa, both with and without S^0^ supplementation. As shown in Figure 1A1, in the absence of S^0^, the SY113 strain exhibited only limited growth under both 0.1 MPa and 27 MPa (corresponding to the strain's isolation depth). The addition of S^0^ significantly promoted the growth of the SY113 strain at atmospheric pressure. Notably, application of HHP further improved the growth. At 0.1 MPa, S^0^ supplementation increased the maximum biomass of the SY113 strain by ~6.5-fold (OD_600nm_: 0.26 ± 0.01 with S^0^ vs. 0.04 ± 0.02 without S^0^). At 27 MPa, the biomass increased by about 11.6-fold (OD_600nm_: 0.58 ± 0.04 with S^0^ vs. 0.05 ± 0.02 without S^0^). Notably, under HHP conditions with S^0^, not only enhanced the growth rate (0.32 ± 0.04 h^−1^ at 0.1 MPa vs. 0.47 ± 0.06 h^−1^ at 27 MPa), but also increased the H_2_S concentrations from 10,062.03 ± 461.81 mmol/OD_600nm_ at 0.1 MPa to 13,118.21 ± 582.30 mmol/OD_600nm_ at 27 MPa (Figure 1B1). These results suggest that HHP promotes the growth of SY113 strain by enhancing H_2_S production.

### 3.2 HHP and S^0^ regulated gene expression of *mbsL* and *mbhL*

**Figure 2:**
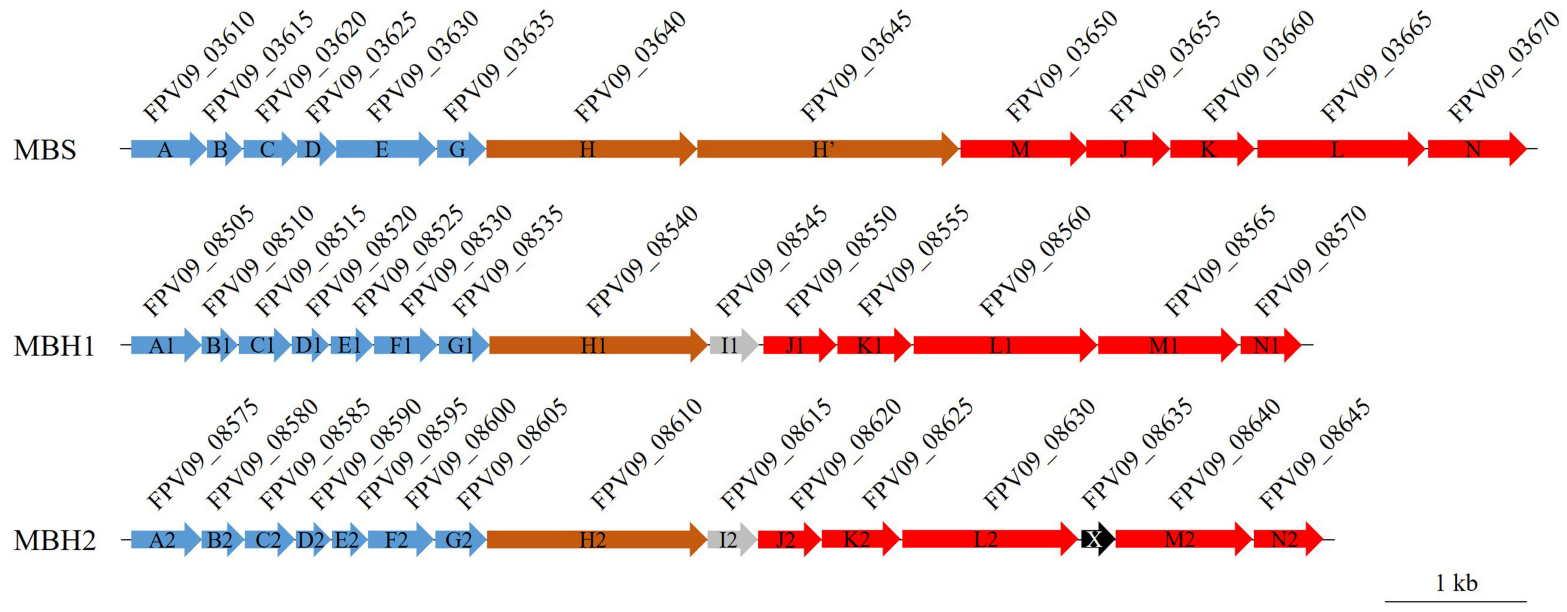
Gene cluster organization of the MBS, MBH1, and MBH2 complexes in SY113 strain. MBS, MBH1, and MBH2 are membrane-bound complexes involved in elemental sulfur or proton reduction. Arrows indicate gene order and transcriptional direction. Genes are color-coded based on function: blue (Na^+^/H^+^ antiporters), brown (proton pumps), red (ferredoxin-dependent oxidation–sulfur reduction modules), and gray/black (proteins of unknown function). The locus tags of genes are displayed above each corresponding gene. Scale bar = 1 kb.

To further evaluate the impact of HHP and S^0^ on the gene expression of SY113 strain, we first analyzed the S^0^ reduction related genes in its genome. As shown in Figure 2, SY113 strain harbors a well conserved *mbs* gene cluster (FPV09_03610 to FPV09_03670), with gene composition and arrangement identical to those of shallow-water model strains. All corresponding subunits exhibited high similarity (average 85.6%) to those of the shallow-sea strain *T. kodakarensis* KOD1 (Supplementary Table 2). In addition, SY113 strain had two copies of *mbh* gene clusters, which were located next to each other on the chromosome. One copy, named as MBH1 (FPV09_08505 to FPV09_08570), showed a high sequence identity (average 73.3%) with shallow-sea strain KOD1, while the other copy, MBH2 (FPV09_08575 to FPV09_08645), possessed a relatively low sequence identity (average 44.5%) (Supplementary Table 2).

Using the catalytic subunit encoding genes *mbsL* (FPV09_03665), *mbhL1* (FPV09_08560) and *mbhL2* (FPV09_08630) as reference, we analyzed the effects of HHP and S^0^ on the transcriptional levels of these gene clusters. As shown in Table 1, the expression of *mbsL* was primarily enhanced by S^0^, while HHP showed no significant regulatory effect. The addition of S^0^ induced the expression of *mbsL* gene at both 0.1 MPa (0.1S/0.1, RQ: 1.92 ± 0.13) and 27 MPa (27S/27, RQ: 4.05 ± 0.25). However, when HHP was applied, no significant expression changes in *mbsL* gene were observed in either S^0^-supplemented (27S/0.1S, RQ: 1.18 ± 0.07) or S^0^-free (27/0.1, RQ: 0.56 ± 0.04) conditions. Notably, combination of S^0^ and HHP produced a synergistic effect, resulting in a 2.26-fold upregulation of *mbsL* gene expression compared to control conditions (27S/0.1, RQ: 2.26 ± 0.14). On the contrary, HHP markedly enhanced the *mbhL1* gene expression irrespective of S^0^ availability (27/0.1, RQ: 2.39 ± 0.03 and 27S/0.1S, RQ: 3.00 ± 0.07). In contrast, S^0^ had no effect on *mbhL1* gene expression (0.1S/0.1, RQ: 0.99 ± 0.02 and 27S/27, RQ: 1.24 ± 0.03). Strikingly, the combined application of S^0^ and HHP synergistically enhanced *mbhL1* gene expression by ~3-fold (27S/0.1, RQ: 2.97 ± 0.03). For another copy of *mbhL2* gene, neither S^0^ nor HHP treatment exerted any significant regulatory effects.

**Table 1:** Expression of genes *mbsL, mbhL1* and *mbhL2* in wild-type under different conditions.

**Gene**	**0.1S/0.1**	**27/0.1**	**27S/0.1S**	**27S/27**	**27S/0.1**
*mbsL*	1.92 ± 0.13	0.56 ± 0.04	1.18 ± 0.07	4.05 ± 0.25	2.26 ± 0.14
*mbhL1*	0.99 ± 0.02	2.39 ± 0.03	3.00 ± 0.07	1.24 ± 0.03	2.97 ± 0.07
*mbhL2*	1.28 ± 0.06	1.09 ± 0.06	1.14 ± 0.06	1.34 ± 0.08	1.47 ± 0.08

0.1 and 27 represent cells were cultured in TRM media under 0.1 MPa or 27 MPa; 0.1S and 27S represent cells were cultured in TRM media supplemented with sulfur under 0.1 MPa or 27 MPa. The values indicate gene expression fold changes (relative quantification).

We further analyzed the transcriptional abundance of these genes under different conditions by means of absolute quantitative RT-PCR. As shown in Supplementary Figure 1, the transcript abundance of *mbhL1* gene at 0.1 MPa was modestly elevated relative to *mbsL* gene (1.4-fold and 1.9-fold with or without S^0^, respectively). At 27 MPa, this difference became more pronounced, with *mbhL1* gene expression exceeding *mbsL* gene by 3.2-fold and 7.1-fold under S^0^-supplemented and S^0^-free conditions, respectively. In contrast to both *mbsL* gene and *mbhL1* gene, *mbhL2* gene exhibited significantly low transcriptional abundance (10-fold lower) in all tested conditions. Collectively, these findings demonstrate distinct regulatory responses among the three gene clusters, MBS expression was predominantly induced by S^0^, and MBH1 was primarily activated by HHP, while MBH2 cluster remained transcriptionally inert under all tested conditions.

### 3.3 MBS is essential for HHP-promoted growth of SY113 strain

To further investigate the roles of MBS and MBH1 in HHP-enhanced growth, we first inactivated the *mbsL* gene (Supplementary Figure 2A). As illustrated in Figure 1A2, the disruption of the *mbsL* gene significantly impaired the growth of the SY113 strain under S^0^-supplemented conditions, with particularly pronounced inhibition observed at 27 MPa. At 0.1 MPa, the maximum biomass decreased from OD_600nm_ 0.26 to 0.19 and the growth rate decreased from 0.32 h^−1^ to 0.18 h^−1^. While at 27 MPa, the maximum biomass decreased by 5.8 times (OD_600nm_: 0.58 ± 0.04 for wild-type vs. 0.10 ± 0.01 for Δ*mbsL*) and the growth rate decreased by 3.4 times (0.47 ± 0.06 h^−1^ for wild-type vs. 0.14 ± 0.04 h^−1^ for Δ*mbsL*). Thus, the strain's growth phenotype shifted from piezophilic to piezosensitive. Notably, under S^0^-supplemented conditions, the Δ*mbsL* mutant exhibited a pronounced reduction in H_2_S production at both 0.1 MPa and 27 MPa compared to the wild-type strain. Specifically, H_2_S yields decreased from 10,062.03 ± 461.81 mmol/OD_600nm_ (wild-type) to 1,685.89 ± 307.28 nmol/OD_600nm_ (Δ*mbsL*) at 0.1 MPa, and from 13,118.21 ± 582.30 mmol/OD_600nm_ to 3,790.13 ± 865.36 nmol/OD_600nm_ at 27 MPa, representing 76.5% and 68.2% reductions, respectively (Figures 1B1, B2).

Moreover, we further disrupted the *mbhL1* gene to investigate the growth and metabolic profiles of the strain when only the MBS was functional (Supplementary Figure 2B). Surprisingly, compared to the wild-type strain, the Δ*mbhL1* strain exhibited enhanced growth in the presence of S^0^ (Figure 1A3). At 0.1 MPa, the maximum biomass increased by 1.8 times (OD_600nm_: 0.46 ± 0.02 for Δ*mbhL1* vs. 0.26 ± 0.01 for wild-type) and the growth rate increased by 1.6 times (0.50 ± 0.05 h^−1^ for Δ*mbhL1* vs. 0.32 ± 0.04 h^−1^ for wild-type). While at 27 MPa, the maximum biomass increased from 0.58 to 0.65 and the growth rate increased by 1.8 times (0.86 ± 0.1 h^−1^ for Δ*mbhL1* vs. 0.47 ± 0.06 h^−1^ for wild-type). The Δ*mbhL1* strain produced H_2_S concentrations of 5,912.02 ± 1,775.23 mmol/OD_600nm_ and 19,676.19 ± 455.25 mmol/OD_600nm_ at 0.1 MPa and 27 MPa (Figure 1B3), respectively, which were comparable to those of the wild-type strain. These results indicate that MBS alone is sufficient for HHP-enhanced growth.

### 3.4 The expression of MBS and MBH in SY113 strain is correlated

Although HHP stimulated *mbhL1* gene expression, disruption of this gene unexpectedly enhanced the growth of SY113 strain. To resolve this discrepancy, we analyzed the expression patterns of *mbsL* gene and *mbhL2* gene in the Δ*mbhL1* mutant. As shown in Table 2, compared to the wild-type strain, *mbhL1* gene disruption led to significantly elevated *mbsL* gene expression under all tested conditions, except for only a 1.65-fold increase at 0.1 MPa with S^0^ supplementation. Notably, *mbhL2* gene expression was also markedly upregulated in the Δ*mbhL1* mutant, particularly under HHP conditions (Table 2). These results suggest a regulatory link between *mbhL1* gene and the expression of *mbsL* gene and *mbhL2* gene. Furthermore, we examined *mbhL1* gene and *mbhL2* gene expression in the Δ*mbsL* mutant. Similarly, *mbsL* gene disruption resulted in significant upregulation of both *mbhL1* gene and *mbhL2* gene across all tested conditions (Table 2). Collectively, these findings demonstrate that the expression of MBS and MBH genes is interdependent.

**Table 2:** Expression of genes *mbsL, mbhL1* and *mbhL2* in mutants under different conditions.

**Strain**	**Gene**	**0.1 MPa**	**27 MPa**	**0.1 MPa + sulfur**	**27 MPa + sulfur**
*ΔmbhL1*	*mbsL*	3.56 ± 0.13	7.72 ± 0.13	1.65 ± 0.03	4.45 ± 0.20
	*mbhL2*	21.88 ± 0.73	85.90 ± 1.79	6.29 ± 0.27	15.25 ± 0.42
*ΔmbsL*	*mbhL1*	12.10 ± 0.88	7.88 ± 0.21	8.55 ± 0.49	4.04 ± 0.20
	*mbhL2*	9.14 ± 0.82	7.43 ± 0.23	5.56 ± 0.21	3.47 ± 0.21
*ΔsurR*	*mbsL*	4.91 ± 0.07	13.68 ± 0.68	2.85 ± 0.03	3.38 ± 0.18
	*mbhL1*	2.22 ± 0.03	1.86 ± 0.04	1.16 ± 0.03	0.37 ± 0.01
	*mbhL2*	0.76 ± 0.09	0.40 ± 0.03	0.28 ± 0.01	0.25 ± 0.01

0.1 and 27 represent cells were cultured in TRM media under 0.1 MPa or 27 MPa; 0.1S and 27S represent cells were cultured in TRM media supplemented with sulfur under 0.1 MPa or 27 MPa. The values in the table shows RT-qPCR results expressed as fold changes (mutant/wild-type).

### 3.5 The HHP-promoted growth of SY113 strain is independent of SurR regulator

**Figure 3:**
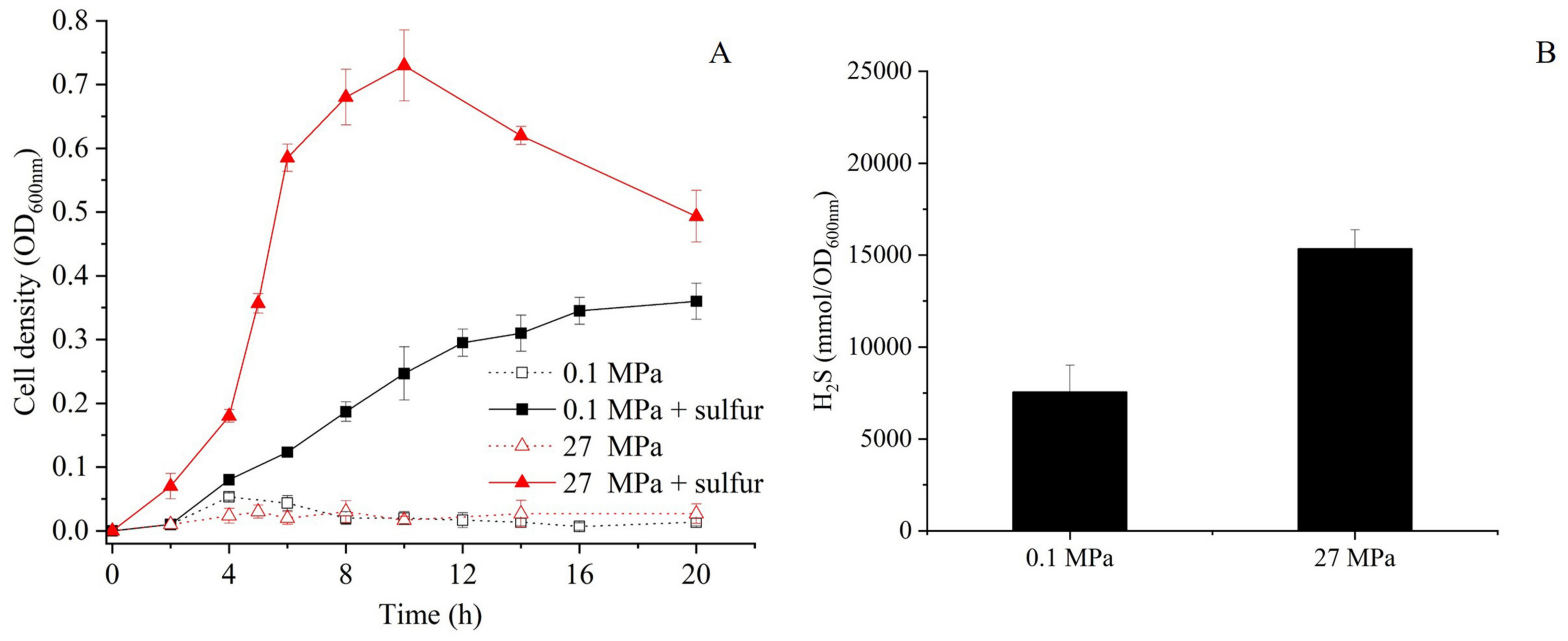
Growth curve and H_2_S production of the Δ*surR* mutants at 0.1 MPa and 27 MPa with or without elemental sulfur. **(A)** Growth curves of Δ*surR* cultured at 0.1 MPa and 27 MPa in the presence or absence of elemental sulfur, measured by OD_600nm_ over time. **(B)** Corresponding H_2_S concentrations for Δ*surR* strain measured after incubation under the same conditions. Black bars represent cultures with sulfur supplementation, error bars represent the standard deviation of three biological replicates. No H_2_S was produced in Δ*surR* mutant in the absence of elemental sulfur.

In shallow-water model strains, sulfur promotes growth by altering the conformation of SurR, which subsequently enhances MBS expression while repressing MBH expression (Lipscomb et al., 2009; Yang et al., 2010). However, how HHP interacts with SurR in SY113 strain is yet to be elucidated. Analysis of the SY113 genome identified a SurR homologous gene (FPV09_04690) flanked by glutaredoxin and ferredoxin, a syntenic region characteristic of Thermococcales. To investigate the potential role of SurR in HHP response, we generated a Δ*surR* mutant (Supplementary Figure 2C). Surprisingly, *surR* gene disruption did not impair the HHP-promoted growth. On the contrary, the Δ*surR* mutant demonstrated superior growth performance under HHP compared to the wild-type strain (Figure 3A). At 0.1 MPa, the Δ*surR* mutant achieved a maximum biomass of OD600 = 0.36 ± 0.03 with a growth rate of 0.38 ± 0.04 h^−1^, whereas at 27 MPa it showed increased maximum biomass (OD_600nm_ = 0.73 ± 0.06) and significantly enhanced growth rate (1.13 ± 0.16 h^−1^). Consistently, H_2_S production under sulfur-supplemented conditions reached 7,561.41 ± 1,448.27 mmol/OD_600nm_ at 0.1 MPa and 15,350.00 ± 1,024.52 mmol/OD_600nm_ at 27 MPa (Figure 3B).

We further analyzed the transcriptional profiles of *mbsL* gene, *mbhL1* gene and *mbhL2* gene following *surR* gene disruption. As shown in Table 2, *surR* gene disruption resulted in upregulated expression of *mbsL* gene under all tested conditions, particularly under HHP. In contrast, *mbhL2* gene exhibited significant expression downregulation in S^0^-supplemented conditions upon *surR* gene disruption. Disruption of the *surR* gene also resulted in a slight expression upregulation of *mbhL1* gene at 0.1 MPa (RQ: 2.22 ± 0.03), while causing a slight downregulation under 27 MPa with S^0^ supplementation (RQ: 0.37 ± 0.01). No significant changes in expression were observed under the other two tested conditions. These findings demonstrate that SurR does not directly regulate either HHP-promoted growth or elemental sulfur reduction.

## 4 Discussion

S^0^ reduction for ATP generation represents a crucial energy metabolism pathway in *Thermococcus*. While studies on shallow-water model strains have described their S^0^ reduction pathway and regulatory mechanisms, the corresponding pathways and regulatory networks in deep-sea *Thermococcus* strain remain poorly characterized. Members of the *Thermococcus* genus typically possess two energy metabolic complexes MBH and MBS. The former reduces protons to generate H_2_, while the latter reduces elemental sulfur to produce H_2_S. In this study, we observed strikingly different HHP responses between these two metabolic pathways (Figure 1). The Δ*mbsL* mutant exhibited significantly reduced H_2_S production but markedly increased H_2_ production, accompanied by impaired growth under HHP (Figure 1, Supplementary Figure 3). Conversely, the Δ*mbhL1* mutant nearly ceased H_2_ production while showing enhanced H_2_S generation, with improved HHP growth performance (Figure 1, Supplementary Figure 3). These findings demonstrate that sulfur reduction confers superior pressure tolerance. Further analysis revealed that the MBS complex contains two proton-pumping subunits (MbsH and MbsH'), compared to just one (MbhH) in MBH (Figure 2), theoretically doubling the energy production efficiency of MBS relative to MBH (Yu et al., 2021). This may explain why the Δ*mbsL* mutant displayed greater pressure sensitivity than the Δ*mbhL1* mutant.

Moreover, for the Δ*mbhL1* mutant, there was no significant difference in H_2_S production compared to the wild-type strain at 0.1 MPa (*P* > 0.05), but it was significantly higher than that of the wild-type strain at 27 MPa (*P* < 0.001). Correspondingly, in the presence of S^0^, the expression level of *mbsL* gene in the Δ*mbhL1* strain showed no significant difference at 0.1 MPa compared to the wild-type strain (RQ: 1.65 ± 0.03), but was significantly upregulated at 27 MPa (RQ: 4.45 ± 0.20). However, the mechanism by which HHP enhances *mbsL* gene expression remains unclear. Similarly, in the Δ*surR* strain, *mbsL* gene expression was significantly upregulated at 27 MPa with S^0^ supplementation, indicating that SurR is not involved in the HHP regulation. These findings suggest that other regulatory factors may be responsible for HHP-dependent *mbsL* gene expression regulation.

The gene expression profile of SY113 strain revealed that the addition of S^0^ did not significantly alter the transcriptional levels of either *mbsL* gene (0.1S/0.1, RQ: 1.92 ± 0.13) or *mbhL1* gene (0.1S/0.1, RQ: 0.99 ± 0.02) at 0.1 MPa. This regulatory pattern is significantly different from that in the extensively studied shallow strains *P. furiosus* (Schut et al., 2007) and *T. kodakarensis* (Santangelo et al., 2011). S^0^ significantly enhanced H_2_S production of the SY113 wild-type strain, while the transcription of *mbsL* gene did not show obvious upregulation. This indicates that the expression of *mbsL* gene is not regulated by SurR and remains at a high expression level even in the absence of S^0^. Analysis of the promoter region of *mbs* cluster in the SY113 strain revealed that, although it contains two SurR-binding consensus sequence (SBS) like the KOD1 strain, the middle three bases of the SBS differ (Supplementary Figure 4). Therefore, we hypothesize that one possible explanation is that the variation in the SBS bases prevents SurR from binding, while another possibility is that the SurR protein sequence in the SY113 strain differs from that in the KOD1 strain, rendering it unable to bind these SBS. Both scenarios would result in the high expression of *mbs* even without S^0^ supplementation. Further comparison of SurR sequences across different *Thermococcus* strains revealed that their N-terminal region and C-terminal region are relatively conserved, whereas the linker region exhibits low homology (Supplementary Figure 5). Differences in the linker region may alter the structure of SurR in the SY113 strain compared to KOD1 strain, affecting its binding to SBS.

As for *mbhL1* gene, its expression abundance was consistently higher than that of *mbsL* gene regardless of the presence of S^0^ (Supplementary Figure 1), suggesting that S^0^ cannot regulate its gene expression through SurR. This regulatory mechanism is distinct from the SurR-dependent regulation observed in shallow-sea strains. In addition, the transcriptional disparity between *mbhL1* gene and *mbsL* gene was more pronounced under high-pressure conditions, suggesting that high pressure also contributes to their transcriptional regulation. However, disrupting *surR* gene significantly suppressed *mbhL1* gene transcription under HHP in the presence of elemental sulfur and completely inhibited H_2_ production (Supplementary Figure 3), indicating that expression of the *mbhL1* gene depends on SurR regulation. A similar phenomenon was observed in the *T. barophilus* MP strain, where the expression level of the *mbh1* was significantly suppressed after the knockout of the *surR* gene (Moalic et al., 2025). Analysis showed that the *mbh1* promoter region contains two SBS, one located at upstream of the BRE/TATA and the other downstream (Supplementary Figure 4). We speculate that SurR binds to the upstream SBS to activate transcription regardless of the presence of S^0^ but cannot bind to the downstream SBS, resulting in the consistently high transcriptional level of *mbhL1* gene. This regulatory pattern resembles the SurR-mediated regulation of ferredoxin: NADP^+^ oxidoreductases 2 (FNOR2) gene expression in *T. kodakarensis*. It showed that a proportion of reduced form of SurR persists even when S^0^ is supplemented in the culture medium. The reduced form of SurR maintains the ability to bind SBS in the FNOR2 promoter region, thereby activating its gene expression (Hidese et al., 2017).

Additionally, the SY113 strain harbors another copy of MBH. Disruption of *mbhL1* gene led to a significant upregulation of *mbhL2* gene, suggesting functional complementarity between the two systems. Therefore, we hypothesize that possessing dual MBH systems may enhance the strain's adaptability to deep-sea environments. Deep-sea microbial adaptation to HHP frequently involves metabolic pathway redundancy. A notable example is the deep-sea *Shewanella piezotolerant* WP3, which maintains duplicate periplasmic nitrate reductases systems (Li et al., 2018) and dimethyl sulfoxide reductase systems (Xiong et al., 2016), conferring exceptional pressure tolerance. We further analyzed 29 published Thermococcus genomes and found that 13 strains possess dual MBH systems (Supplementary Figure 6). Among these 13 strains, 12 clustered into two distinct evolutionary clades. One clade comprised 7 strains, all isolated from deep-sea hydrothermal vents. The other clade contained 5 strains: 2 from deep-sea hydrothermal vent, 2 from high temperature and high-pressure oil reservoirs, and was derived from a shallow submarine thermal spring. These results suggest that possessing dual MBH systems may facilitate these strains' adaptation to extreme high-temperature and/or high-pressure environments. It is noteworthy that HHP affects multiple aspects of cells, such as membrane fluidity, biomacromolecule function, and energy metabolic pathways (Oger and Jebbar, 2010). Correspondingly, microbial adaptation to HHP environments is not determined by a single factor but rather results from the combined effects of multiple factors.

## 5 Conclusion

Our findings revealed that the MBS-mediated elemental sulfur reduction pathway is important for the SY113 strain's adaptation to deep-sea HHP environments. Moreover, we found significant differences in the regulatory patterns of MBS and MBH between the deep-sea strain SY113 and shallow-water strains in response to elemental sulfur. Notably, HHP also modulates the expression of MBS and MBH, mirroring observations in other deep-sea piezophiles such as *T. piezophilus* and *T. barophilus*. This research uncovers the diversity of sulfur metabolism regulation in deep-sea *Thermococcus* species and expands our understanding of their HHP adaptation mechanisms.

## Data Availability

The original contributions presented in the study are included in the article/Supplementary material, further inquiries can be directed to the corresponding author.
