# Supplementary material for: High hydrostatic pressure enhanced the growth of deep-sea Thermococcus aciditolerans by promoting the reduction of elemental sulfur
Source: Front Microbiol. 2025 Aug 18;16:1643593. doi: 10.3389/fmicb.2025.1643593 (PMC12401011; doi:10.3389/fmicb.2025.1643593)
Supplement: Supplementary file 1 [file Supplementary_file_1.docx]

Supplementary Material

High hydrostatic pressure enhanced the growth of deep-sea *Thermococcus aciditolerans* by promoting the reduction of elemental sulfur

Ze-Xi Jiao^1,2^, Xue-Gong Li^1,3,4,^*, Wei-Jia Zhang^1,3,4^, Guan-Yuan Zhang^1,2^, Shi-Jie Bai^5^, Ling Fu^6^ and Long-Fei Wu^3,7^

^1^Laboratory of Deep-Sea Microbial Cell Biology, Institute of Deep-sea Science and Engineering, Chinese Academy of Sciences, Sanya, PR China

^2^University of Chinese Academy of Sciences, Beijing, PR China

^3^International Associated Laboratory of Evolution and Development of Magnetotactic Multicellular Organisms, CNRS-Marseille/CAS-Sanya

^4^Institution of Deep-sea Life Sciences, IDSSE-BGI, Hainan Deep-sea Technology Laboratory, Sanya, Hainan, PR China

^5^Laboratory of Marine Viruses and Molecular Biology, Institute of Deep-sea Science and Engineering, Chinese Academy of Sciences, Sanya, PR China

^6^State Key Laboratory of Proteomics, Beijing Proteome Research Center, National Center for Protein Sciences• Beijing, Beijing Institute of Lifeomics, Beijing, China

^7^Aix Marseille Univ, CNRS, LCB, IMM, IM2B, Marseille, France

*** Correspondence:**Corresponding Author: Xue-Gong Li
[xuegongli@idsse.ac.cn](mailto:xuegongli@idsse.ac.cn)

# Supplementary Figures


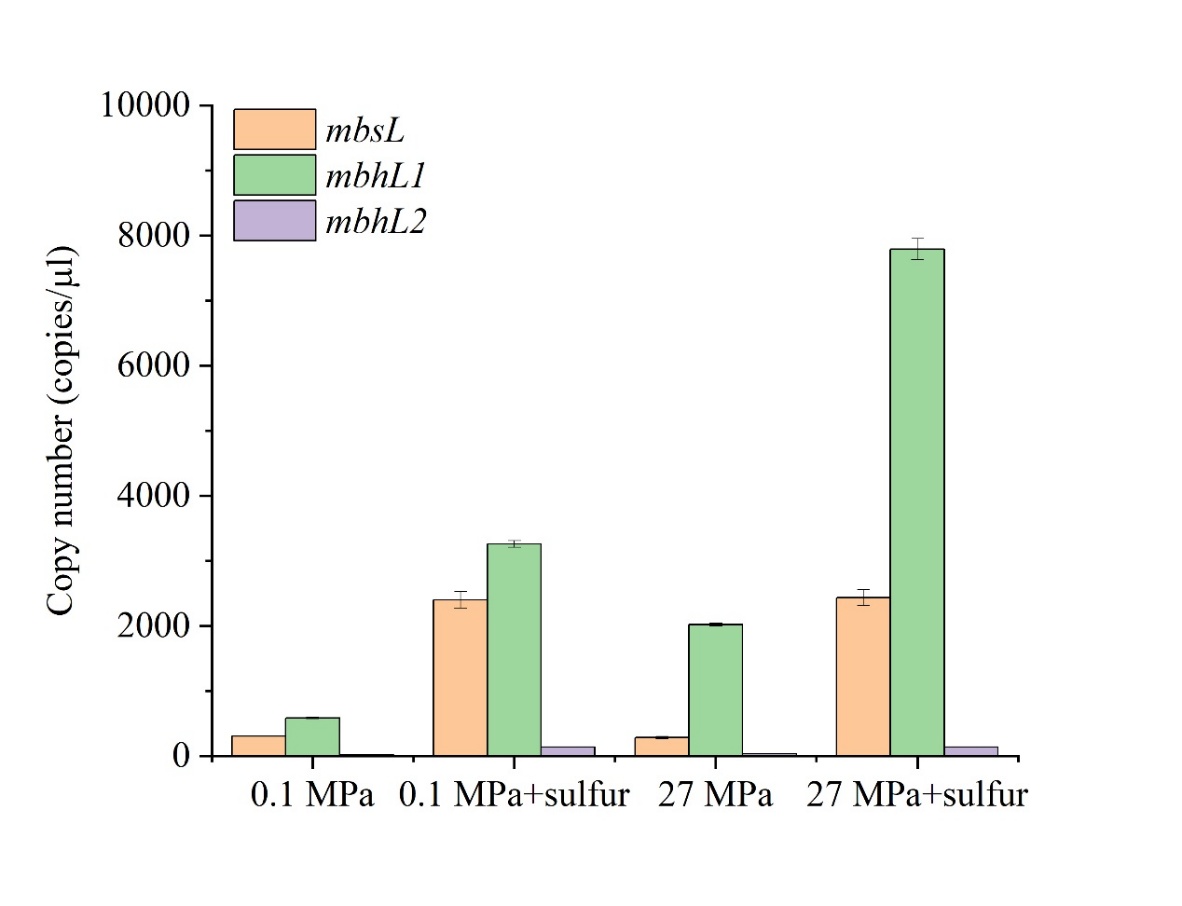


**Figure S1. Expression levels of *mbsL*, *mbhL1* and *mbhL2* in SY113 strain under different conditions.** The copy number of *mbsL*, *mbhL1* and *mbhL2* in SY113 cells cultivated under different conditions was determined by absolute quantitative RT-PCR. Each value is the average of three measurements.


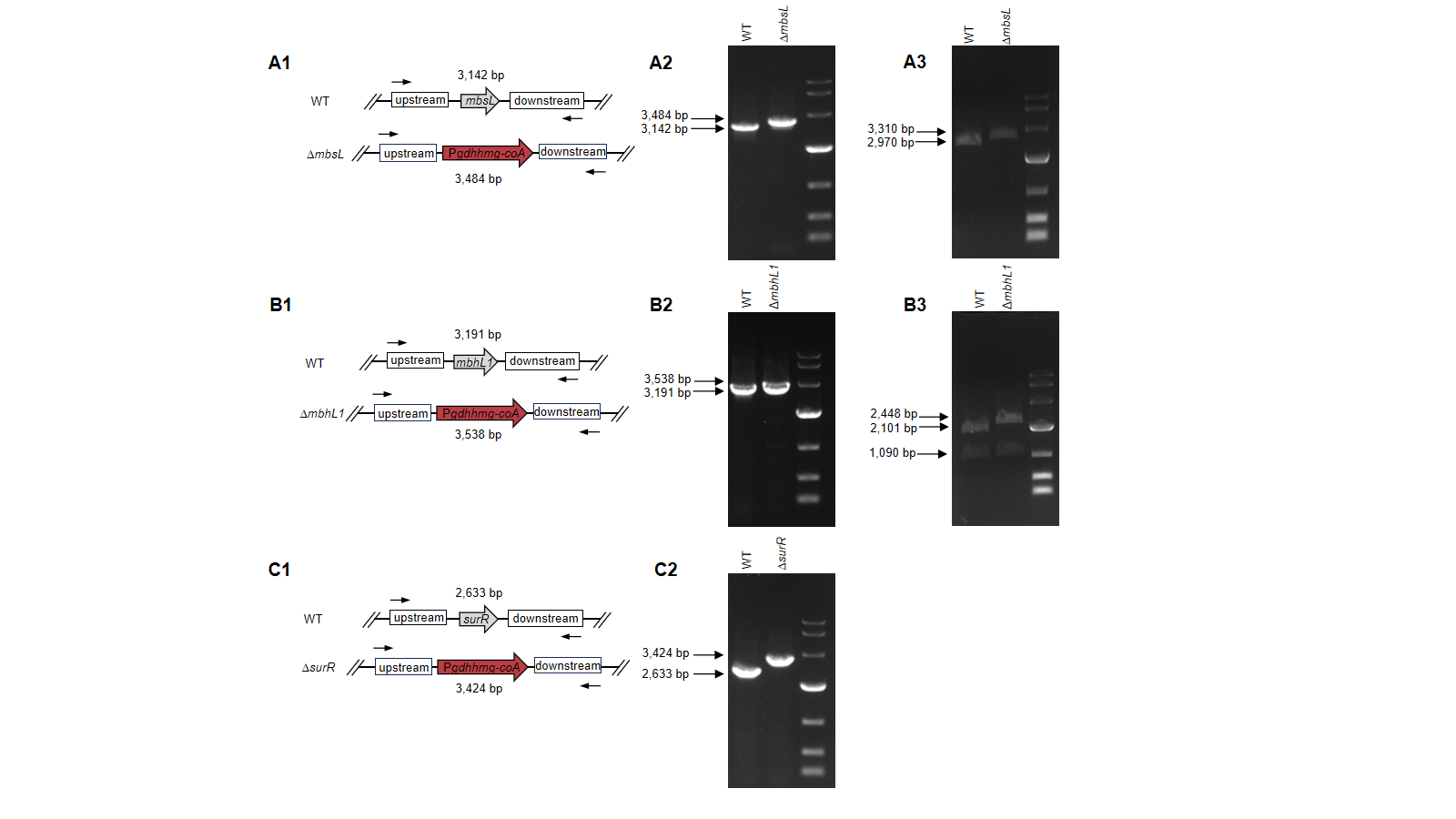


**Figure S2. PCR Confirmation of Gene Disruptions: Δ*mbsL*, Δ*mbhL1* and Δ*surR*.**

(A) Verification of *mbsL* disruption. (A1) Schematic illustration of the expected PCR products for WT and Δ*mbsL* strains. (A2) PCR analysis shows a 3,484 bp band for WT and a 3,142 bp band for Δ*mbsL*. (A3) *ScaⅠ* digestion of the PCR product for further validation. Digestion of the WT product shows bands of 3,310 bp, while digestion of the Δ*mbhL1* product shows bands of 2,970 bp. (B) Verification of *mbhL1* disruption. (B1) Schematic illustration of expected PCR products for WT and Δ*mbhL1* strains. (B2) PCR analysis shows a 3,538 bp band in WT and a 3,191 bp band in Δ*mbhL1*. (B3) *ScaⅠ* digestion of the PCR product for further validation. Digestion of the WT product shows bands of 2,101 bp and 1,090 bp, while digestion of the Δ*mbhL1* product shows bands of 2,448 bp and 1,090 bp. (C) Verification of *surR* disruption. (C1) Schematic illustration of expected PCR products for WT and Δ*surR* strains. (C2) PCR analysis shows a 3,424 bp band in WT and a 2,633 bp band in Δ*surR*.


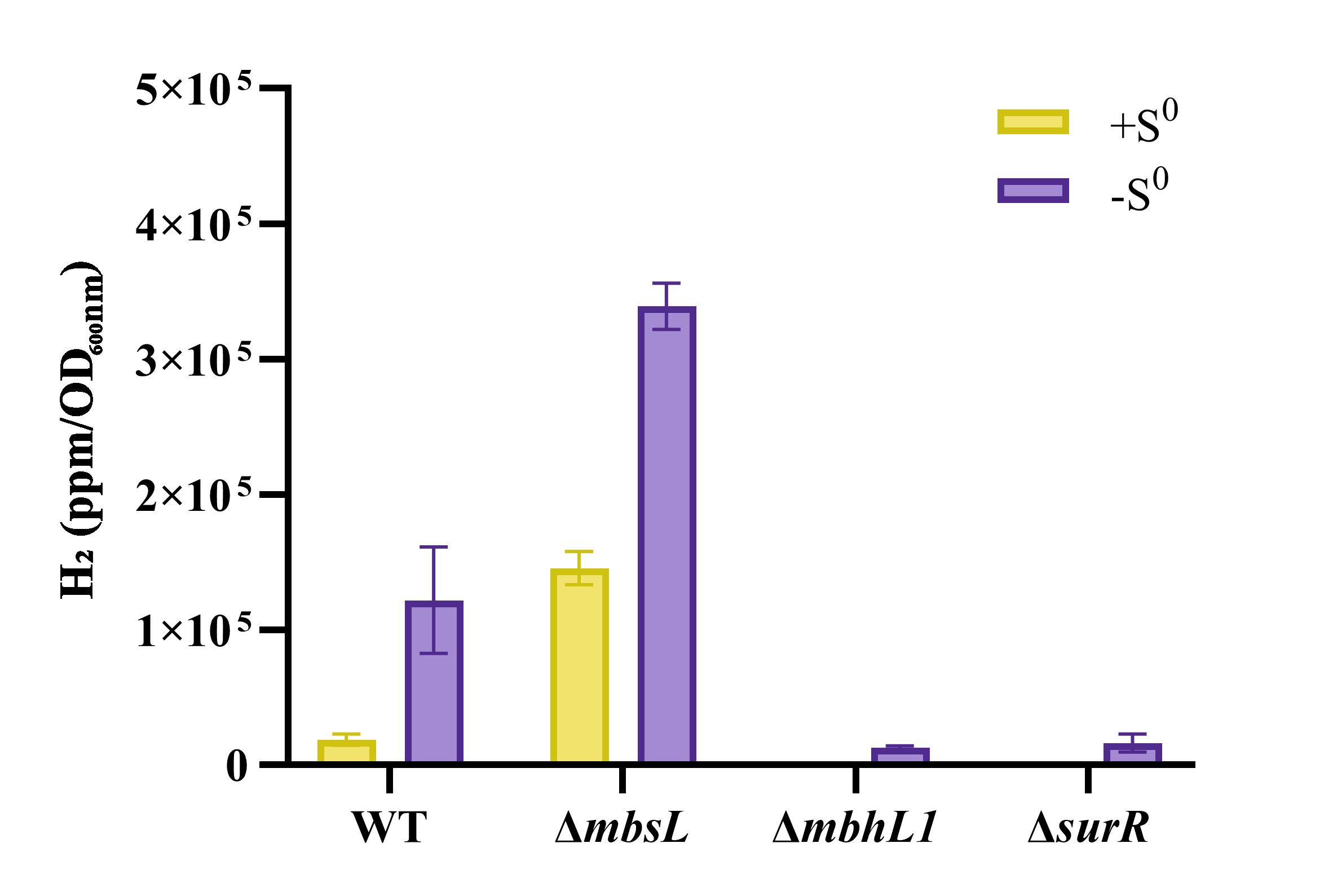


**Figure S3. Hydrogen (H₂) production in wild-type and mutant strains in the presence and absence of elemental sulfur.**

H₂ concentrations (ppm) were measured in the headspace of cultures of wild-type (WT), Δ*mbsL*, Δ*mbhL1* and Δ*surR* strains grown with (+S⁰, yellow bars) or without elemental sulfur (–S⁰, purple bars). Data represent three independent biological replicates' mean ± standard deviation (SD).


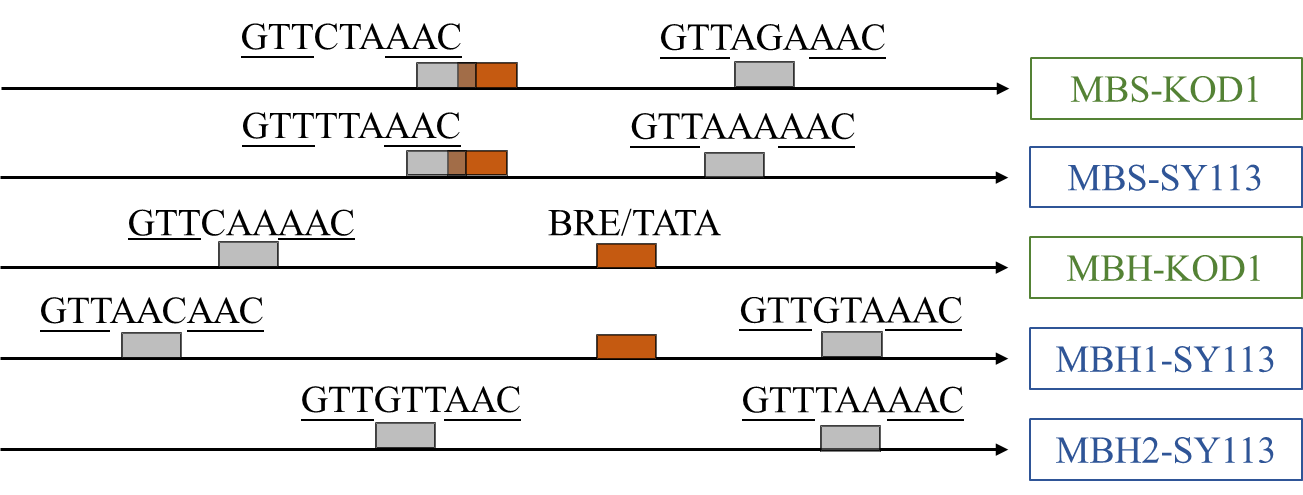


**Figure S4. Schematic representation of the *mbs*, *mbh1*, and *mbh2* promoter regions in SY113 strain and KOD1 strain.**

Orange boxes indicate BRE/TATA promoter elements; grey boxes represent SurR-binding consensus sequences (SBS); black arrows indicate transcriptional direction. Promoter element positions are as follows: in SY113, the *mbs* (FPV09_03610–03670) BRE/TATA box is located at 658,808 bp – 658,822 bp, SBS1 at 658,814–658,822, and SBS2 at 658,857 bp – 658,862 bp; *mbh1* (FPV09_08505–08570) BRE/TATA at 1,547,148 bp – 1,547,162 bp, SBS1 at 1,547,102 bp – 1,547,110 bp, and SBS2 at 1,547,215 bp – 1,547,223 bp; *mbh2* (FPV09_08575–08645) SBS1 at 1,555,520 bp – 1,555,528 bp, and SBS2 at 1,555,624 bp – 1,555,632 bp. In KOD1, the *mbs* (TK1214–1226) BRE/TATA box is located at 1,075,161 bp – 1,075,174 bp and SBS1 at 1,075,118 bp – 1,075,126 bp; the *mbh* (TK2080–2093) BRE/TATA box is located at 1,871,309 bp –1,871,321 bp and SBS at 1,871,299 bp – 1,871,307 bp.


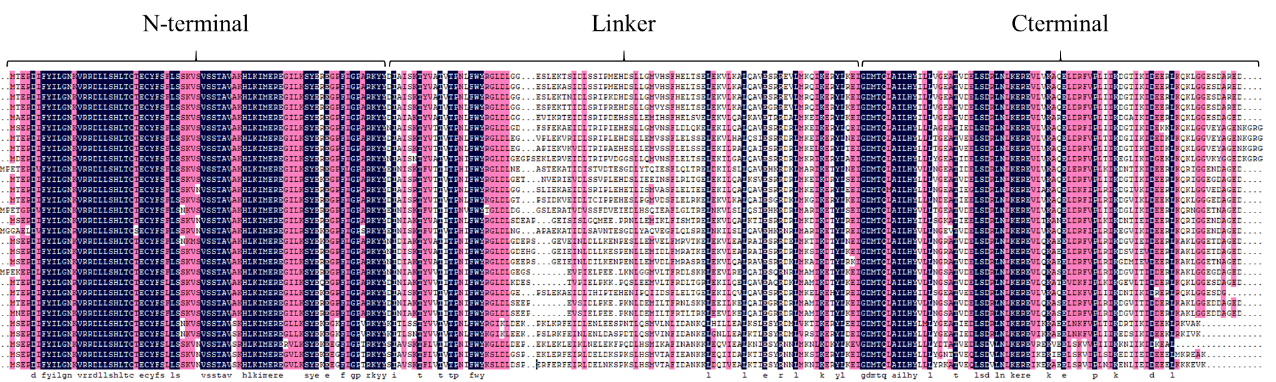


**Figure S5. Alignment of SurR sequences from 29 *Thermococcus.***


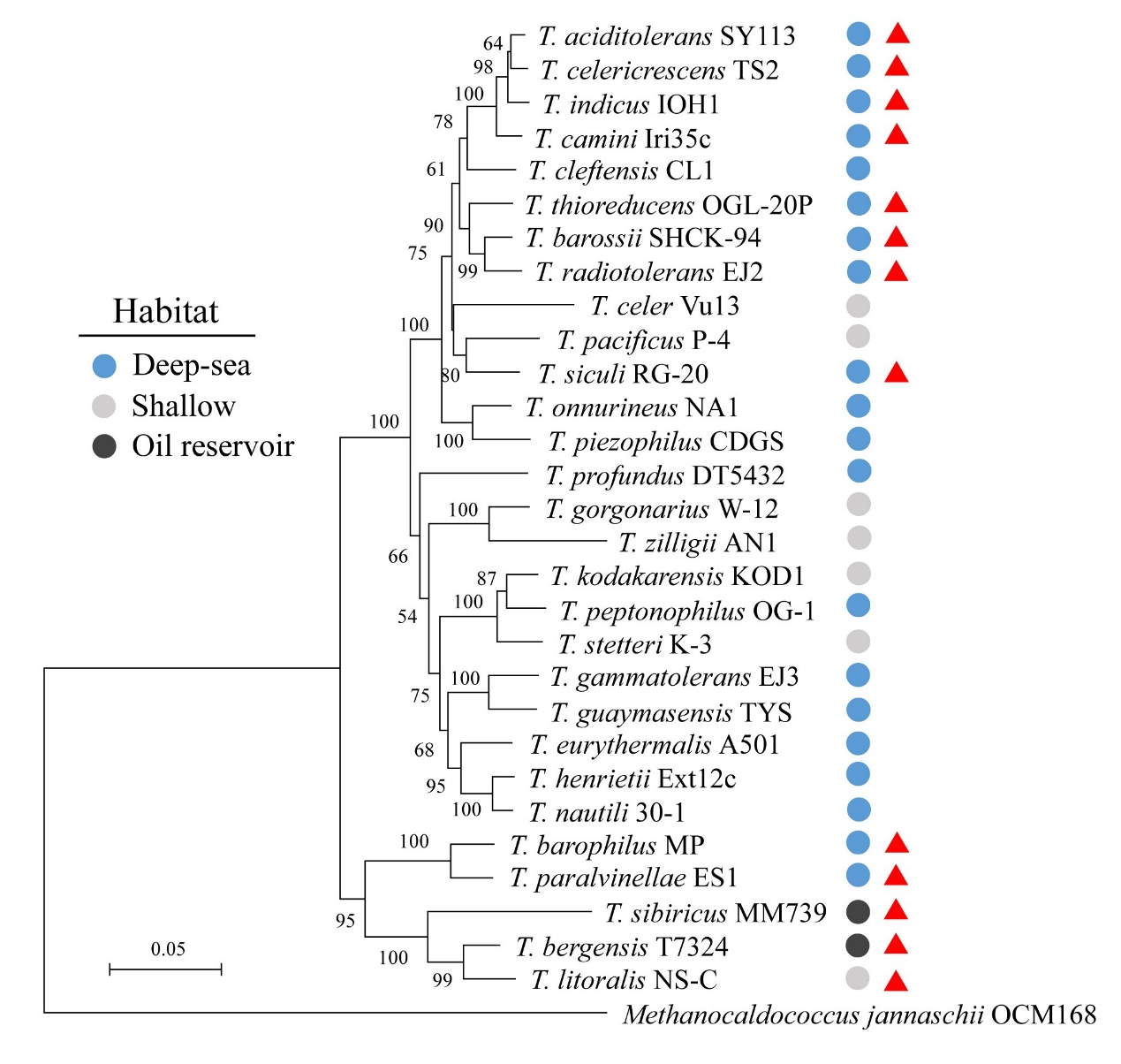


**Figure S6. Neighbor-joining phylogenetic tree of 29 *Thermococcus*.**

The phylogenetic tree based on the concatenated 120 microbial marker proteins using the MEGA version 11. The evolutionary distances were computed using the p-distance method and are in the units of the number of amino acid differences per site. Bootstrap values above 50 % based on 1,000 replicates are shown at nodes. Bar, number of amino acid differences per site. Red triangles indicate strains harboring two sets of MBH.

**Table S1. All primers used in this research.**

| **Name** | **sequences（5’-3’）** |
| --- | --- |
| plasmid-F | TCGTAATCATGGTCATAGCTGTTTCCTGTG |
| plasmid-R | GGCATGCAAGCTTGGCACTGGCCG |
| *por*-F | CGAGAAGAGCAAGGAGGAG |
| *por*-R | GGATCGAGGTGTTGGTGAT |
| 04690-up-F | CAGTGCCAAGCTTGCATGCCGCACATTAACACGGAGATG |
| 04690-up-R | **TTTTCAA**GACCCACACCACCCAAATC |
| 04690-P*gdh*-F | **TGGGTC**TTGAAAATGGAGTGAGCTGA |
| 04690-hmg-coA-R | **CATCGCT**TCATCTCCCAAGCATTTTATGAG |
| 04690-dw-F | **GAGATGA**AGCGATGCCAGAGAAGAT |
| 04690-dw-R | **AGACCCT**CTTGACGAGTTCCTTGACA |
| 04690-hhp-tac-F | **CGTCAAG**AGGGTCTTCCTCATCTCGG |
| 04690-hhp-tac-R | **GCTATGACCATGATTACGA**AGCGATATATTTATATAGGG |
| 03665-up-F | CAGTGCCAAGCTTGCATGCCTCAAGCAGTTCGACAAGTA |
| 03665-up-R | **TTTTCAA**TATCCTCACCTCATATCACG |
| 03665- P*gdh* -F | **GAGGA**TATTGAAAATGGAGTGAGCTG |
| 03665-hmg-coA-R | **TGTCCGG**TCATCTCCCAAGCATTTTATGAG |
| 03665-dw-F | **GAGATGA**CCGGACATAGACAGGTGAT |
| 03665-dw-R | **AGACCCT**CGAACGATGGTATGGACAG |
| 03665-hhp-tac-F | **TCGTTCG**AGGGTCTTCCTCATCTCGG |
| 03665-hhp-tac-R | **GCTATGACCATGATTACGA**AGCGATATATTTATATAGGG |
| 08560-up-F | CAGTGCCAAGCTTGCATGCCGAGGAGGTGAGATGAATGG |
| 08560-up-R | **TTTTCAA**CGAGCGTGATGATGAACT |
| 08560- P*gdh* -F | **ACGCTCG**TTGAAAATGGAGTGAGCTG |
| 08560-hmg-coA-R | **ACTTCGG**TCATCTCCCAAGCATTTTATGAG |
| 08560-dw-F | **GAGATGA**CCGAAGTTGTTGGAGTAGG |
| 08560-dw-R | **AGACCCT**TGGAAGAGCAGGTCTATGA |
| 08560-hhp-tac-F | **TCTTCCA**AGGGTCTTCCTCATCTCGG |
| 08560-hhp-tac-R | **GCTATGACCATGATTACGA**AGCGATATATTTATATAGGG |
| 03610-F1 | **CGGCCAGTGCCAAGCTTGCATGCC**CTGCTGTCGCTCATAGTC |
| 03610-R1 | **CAGCTATGACCATGATTACGA**GTACTCATCTTCGTCAACATC |
| 03665-F1 | **CGGCCAGTGCCAAGCTTGC**ATGCCATGAGCGAGAGGAAGAATG |
| 03665-R1 | **CAGCTATGACCATGATTACGA**CGTGATGAGGAACAGGTC |
| 08505-F1 | **CGGCCAGTGCCAAGCTTGCATGCC**CACGACGAAGGACATCAT |
| 08505-R1 | **CAGCTATGACCATGATTACGA**TTGAGTTGCTGAGGATTGT |
| 08560-F1 | **CGGCCAGTGCCAAGCTTGCATGCC**GAGGATATGCGGAATCTGT |
| 08560-R1 | **CAGCTATGACCATGATTACGA**CGATGGTGTTGATGGAGTA |
| 03665-F | GTTCTGGATAATGGGCTTTG |
| 03665-R | CCGGGTATGTCCCTTCTG |
| 08560-F | ATAGGCTACGACACCACCC |
| 08560-R | CCTGATGTGCTTCTCCTCC |
| 08630-F | TCGCCTGGCTTTCTGTTA |
| 08630-R | TCGCCTGGCTTTCTGTTA |

Note: Bold and underlined indicate overlapping sequences, where bold indicates additional sequences added at the 5 'end of the primer.

**Table S2. Sequence identity (%) of individual subunits of MBS, MBH1, and MBH2 between *T. aciditolerans* SY113 and *T. kodakarensis* KOD1.**

| MBS | Identity (%) | MBH1 | Identity (%) | MBH2 | Identity (%) |
| --- | --- | --- | --- | --- | --- |
| MbsA | 81.4 | MbhA1 | 72.0 | MbhA2 | 57.4 |
| MbsB | 88.1 | MbhB1 | 80.3 | MbhB2 | 43.6 |
| MbsC | 84.7 | MbhC1 | 78.9 | MbhC2 | 53.5 |
| MbsD | 88.3 | MbhD1 | 81.8 | MbhD2 | 51.3 |
| MbsE | 84.3 | MbhE1 | 80.6 | MbhE2 | 45.8 |
| MbsG | 85.1 | MbhF1 | 86.4 | MbhF2 | 43.9 |
| MbsH | 77.8 | MbhG1 | 80.7 | MbhG2 | 46.7 |
| MbsH’ | 82.0 | MbhH1 | 75.5 | MbhH2 | 44.2 |
| MbsM | 92.9 | MbhI1 | 63.5 | MbhI2 | 26.4 |
| MbsJ | 89.6 | MbhJ1 | 81.0 | MbhJ2 | 58.5 |
| MbsK | 86.9 | MbhK1 | 50.9 | MbhK2 | 33.2 |
| MbsL | 95.2 | MbhL1 | 70.6 | MbhL2 | 46.6 |
| MbsN | 76.7 | MbhM1 | 67.6 | MbhM2 | 36.0 |
|  |  | MbhN1 | 57.6 | MbhN2 | 35.5 |

Note: Each subunit of the MBS, MBH1, and MBH2 complexes from *T. aciditolerans* SY113 was compared to its homolog in *T. kodakarensis* KOD1. Since KOD1 contains only a single *mbh* operon, both MBH1 and MBH2 subunits from SY113 were independently aligned to this sole MBH complex.
